# Intelligent structural microbatteries for adaptive microrobots

**DOI:** 10.1093/nsr/nwaf467

**Published:** 2025-10-30

**Authors:** Jiaxin Ma, Sang-Young Lee, Zhong-Shuai Wu

**Affiliations:** State Key Laboratory of Catalysis, Dalian Institute of Chemical Physics, Chinese Academy of Sciences, China; School of Materials Science and Engineering, Zhengzhou University, China; Department of Chemical and Biomolecular Engineering, Yonsei University, Republic of Korea; State Key Laboratory of Catalysis, Dalian Institute of Chemical Physics, Chinese Academy of Sciences, China; Dalian National Laboratory for Clean Energy, Chinese Academy of Sciences, China

## Abstract

This Perspective envisions intelligent structural microbatteries that integrate energy, structure and adaptive control, enabling microrobots to operate autonomously and efficiently in extreme environments.

Microrobots at millimeter to sub-millimeter scales are poised to revolutionize strategic domains including precision medicine, environmental sensing and deep-space exploration [1]. However, these transformative platforms remain fundamentally constrained by power supply systems that fail to meet the combined demands of miniaturization, adaptability and intelligence [2]. Traditional batteries separated from mechanical and structural roles, are intrinsically incompatible with the form, function and autonomy required by microrobots [3]. A paradigm shift is urgently required toward structural batteries that integrate energy, structure and function into adaptive subsystems co-evolving with microrobot behavior. Unlike deformable or stretchable microbatteries, structural microbatteries simultaneously deliver electrochemical performance and mechanical support as integral elements of the microrobot skeleton. Specifically, the electrodes, current collectors and encapsulants within structural microbatteries are designed to act as stress-bearing components, enabling the entire device to sustain compressive and bending deformation while maintaining its electrochemical functionality. Miniaturization to the millimeter and sub-millimeter scales introduces distinctive challenges in fabrication and integration. Conventional electrode particles are typically too coarse for miniaturized systems, necessitating nanoscale or hierarchical architectures to ensure continuous electronic pathways. The processes of slurry casting and electrolyte infiltration require micrometer-level precision, whereas device encapsulation depends on ultrathin conformal barriers rather than conventional bulky sealing layers. Addressing these challenges is crucial for realizing structural microbatteries that integrate energy storage with mechanical load-bearing functions in microrobots. In this perspective, we discuss the emerging microscale power supply systems for long-endurance microrobots, such as structural and shape-adaptable microbatteries, self-reconfigurable and self-sustained energy systems. Furthermore, we propose key design principles, highlight multifunctional architectures, and outline future directions toward

compact, adaptive and mission-aware energy systems essential for autonomous microrobot platforms.

Structural microbatteries featuring non-standard and shape-adaptive geometries provide a foundational strategy for embedding electrochemical energy storage devices directly into microrobot structures. Recent lithium-ion microbatteries have demonstrated energy density exceeding 10 mWh cm^−2^, supporting microrobot operations for up to 30 minutes. Nevertheless, substantial progress in structural adaptability and energy density remains essential to satisfy the requirements of long-term operation and conformal integration with dynamically deformable microrobot platforms. Representative performance metrics are summarized in Fig. 1a, which illustrates the complementary advantages of 3D printing and laser etching in device fabrication.

**Figure 1.**
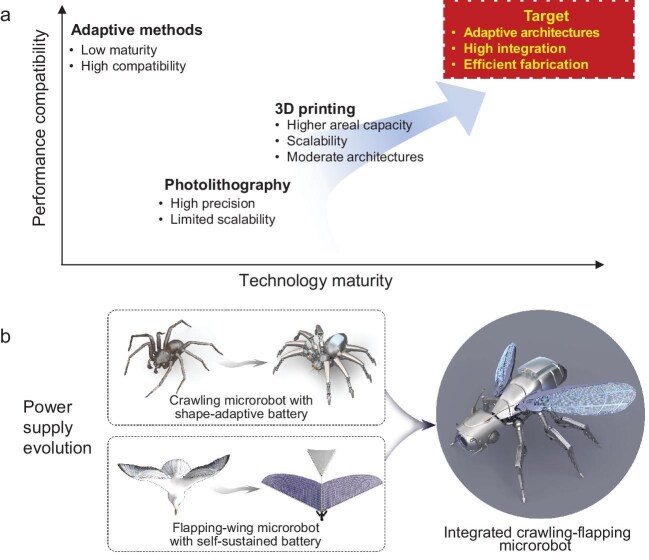
Architected power supply systems for adaptive, intelligent and long-endurance microrobots: (a) fabrication methods, (b) power supply evolution.

Shape-conformable microbatteries fabricated by high-resolution 3D printing and laser lithography can adopt the geometries (e.g. gyroid, mesh, branched) that fit seamlessly within the internal frameworks of microrobots. By incorporating interfacial adhesion modifiers, printable formulations and conformal nanoscale encapsulation layers, these microfabrication strategies enable compatibility with heterogeneous substrates, facilitate electrolyte integration, and support advanced packaging designs, thereby ensuring reliable device integration. This allows power modules to double as structural elements, maximizing utilization of space and reducing inert proportions. Future architectures may go beyond conformation toward transformation, enabling self-morphing batteries that shift geometry to match locomotion modes or environmental feedback. However, miniaturization to the microscale intensifies several intrinsic challenges, including increased ionic resistance arising from the limited electrolyte volume, elevated electrode–electrolyte interfacial impedance, and localized heating induced by higher current densities. These issues are further compounded in irregularly shaped or non-planar microbattery architectures, where geometric asymmetry can induce non-uniform current distributions, localized stress concentrations, and distorted ion transport pathways. Mitigating these issues requires fine control of electrode porosity, uniformity of electrolyte thickness and interface chemistry. Designs such as interdigital microcolumns and porous voxel lattices featuring multi-direction ion transfer ensure high rate performance, supporting rapid actuation demand for dynamic tasks like jumping, flapping, and evasive maneuvers. Moreover, the introduction of gradient porosity and anisotropic ionic/electronic conductivity enables multiscale structural coupling, where localized architectures are spatially programmed to achieve high capacity, high power and mechanical reinforcement in distinct domains [4]. This hierarchical design strategy coordinates micro–nano structural features with macroscopic mechanical demands, establishing an integrated framework for power units with optimized electrochemical and structural performance.

The mechanical compliance of microrobots introduces frequent bending, stretching and compression, calling for energy systems that integrate intrinsic deformability with structural load-bearing functions. Structural microbatteries address this challenge through architectures that maintain both structural and electrochemical integrity (Fig. 1b). Serpentine-shaped current collectors and interconnects help distribute mechanical strain uniformly and facilitate mechanical stress transfer across the device framework [5], while elastomeric polymer binders and soft encapsulants sustain ion transport and electrical connectivity under repeated deformation cycles. By integrating piezo-responsive polymers, strain-adaptive microelectrodes and stretchable ionic-liquid matrices, microbattery can dynamically modulate power output in accordance with microrobot motion, resulting in dynamic stress redistribution and enhanced energy efficiency during active locomotion [6]. To improve morphological adaptability, the incorporation of programmable hydrogels into the battery matrix enables shape-memory and reconfigurable characteristics that can be activated by thermal, electrical, or chemical triggers. These shape-morphing mechanisms offer programmable deformation modes that can be tailored to match microrobot locomotion or respond to environmental stimuli, thereby establishing a foundation for next-generation reconfigurable microscale power supply systems [7]. Beyond generic deformation tolerance, structural microbatteries must be engineered to accommodate specific environmental conditions. For implantable microrobots, biocompatible solid-state or bioresorbable electrolytes are needed to ensure safety and controlled degradation. In aquatic or subterranean microrobots, corrosion-resistant interfaces and hermetic thin-film encapsulation protect against humidity, pressure and chemical attack. For deep-space or polar missions, wide-temperature chemistries and radiation-tolerant barriers mitigate thermal cycling and vacuum exposure. These considerations highlight the practical feasibility of structural microbatteries across biomedical, terrestrial and extraterrestrial platforms.

Exposure to extreme or inaccessible environments places stringent demands on power system durability, thereby driving the evolution of intelligent microbattery systems with autonomous self-healing capabilities. To address persistent mechanical degradation in confined and repair-limited environments, intrinsically self-healing microbattery materials, such as dynamic covalent networks, ionic elastomers, and hydrogel-based electrolytes, are vital to restore both mechanical integrity and electrochemical continuity without external intervention [8]. Rigorous assessment of self-healing requires quantitative indicators such as capacity recovery efficiency, ionic conductivity restoration (typically 80%–95%), and charge-transfer resistance recovery (∼80%–90%), which together demonstrate the effectiveness of electrochemical healing.

Beyond structural resilience, long-term autonomy further depends on the ability of power systems to harvest and respond to ambient energy sources. One promising direction is the integration of photovoltaic harvesting with electrochemical energy storage in a unified architecture with representative solar-to-stored energy efficiency of ∼7%–10% under standard illumination. This approach is particularly suited for microrobot platforms designed to operate in open environments, such as terrestrial, aerial, or extraterrestrial settings, where direct solar irradiation is exploited to enable a sustainable energy source [9]. The key to this integration is the electrochemical compatibility between the solar cells’ output characteristics and the charge acceptance profiles of the lithium-ion microbatteries, ensuring efficient energy transfer and storage under variable illumination conditions. Effective interfacial engineering between the photovoltaic and energy storage layers plays a pivotal role in enhancing energy conversion efficiency and maintaining long-term stability.

While ambient energy harvesting is valuable, energy-isolated environments including subterranean spaces, aquatic systems and implantable biomedical devices, require intrinsically self-sufficient power systems capable of long-term operation without external input. Wireless energy transfer techniques, including resonant inductive coupling, acoustic energy transmission and magnetoelectric coupling, allow power delivery without physical contact thus providing complementary pathways for delivering power through non-transparent or inaccessible media [10]. A major obstacle is the mitigation of energy dissipation across the interfaces coupling reception, conversion and storage units. Achieving monolithic integration of microbatteries with energy-harvesting modules *via* co-fabrication or self-assembly could enable self-rechargeable microrobots capable of sustained operation in surgical, subterranean, and extraterrestrial environments.

While ambient energy harvesting and wireless charging are promising, they alone cannot guarantee resilience. Intelligent energy management transforms passive power systems into adaptive and mission-aware subsystems. Specifically, reinforcement learning models can be trained to dynamically optimize charge–discharge profiles in response to mission conditions, reducing overpotential and minimizing capacity fade. Predictive maintenance algorithms based on voltage/time series analysis can detect early signs of degradation (e.g. SEI thickening, lithium plating), triggering autonomous recovery strategies such as activating self-healing polymer domains. AI-enabled coordination of charging schedules within microrobot swarms can mitigate energy bottlenecks and prolong swarm-level endurance. In this framework, the intelligence is defined as a closed-loop sense–decide–respond process. Structural microbatteries integrate sensing elements to monitor strain, or current fluctuations, providing real-time feedback on microrobot states. Lightweight controllers or distributed algorithms analyze these signals to anticipate energy demand, while the battery adapts by modulating discharge, redistributing loads, or activating self-healing. At the system level, coordination between distributed power units and microrobot control networks ensures energy management, allowing the battery to function as an adaptive, mission-aware subsystem. AI-enhanced microsystems function as adaptive supervisory that optimize energy utilization at the device scale and coordinate power distribution across microrobot networks, thereby extending operational autonomy and enabling emergent collective behaviors.

Taken together, advances in intelligent energy harvesting, wireless transmission and AI-based power control are redefining structural microbatteries as mission-adaptive platforms, unlocking fully autonomous microrobot operation in extreme environments. Future integrated microsystems must seamlessly converge energy storage, mechanical function and intelligent control, representing a fundamental shift from discrete power units to multifunctional and self-adaptive subsystems. This convergence bridges materials science, systems engineering and artificial intelligence, empowering microrobots with unprecedented levels of autonomy, adaptability and mission resilience. Structural microbatteries embody a functional convergence of energy storage and structural reinforcement, establishing them as indispensable power systems for the next generation of application-constrained microrobots.
